# Annotations

**Published:** 1909-07-24

**Authors:** 


### ANNOTATIONS.

### Medical Jargon.

At the annual dinner of the Medico-Legal Society held at the Holborn Restaurant on July 13 under the presidency of Mr. Justice Walton, the toast of "The Law and Medicine" was proposed by Sir W. J. Collins, who, in the course of his remarks, pleaded for the use of simpler language in medical and scientific papers. The Pathological Society came under his especial disapprobation on account of the incomprehensible jargon of scientific slang used at its meetings, which should, in his judgment, be replaced by the Anglo-Saxon tongue. If Sir William means that grammar is neglected and sense obscured by the careless and incorrect use of contractions and elliptical constructions in scientific works, then he has undoubtedly put his finger on a very weak spot in modern medical literature. But if he wants Anglo-Saxon words or words of Anglo-Saxon derivation to replace the compound Greek and Latin terms which are almost entirely used in Great Britain to express new scientific ideas, we fear his appeal will not be of very much avail. With the example of such stylists as Tyndall and Huxley it can never be contended that scientific writing need be either slovenly or barbarous, notwithstanding the occasional cumbersomeness of the technical terms in use. More stringent requirements of candidates for registration as medical students, with a view to ensuring some knowledge of, and therefore some respect for, their mother tongue, would be much more likely to obviate the tendencies to which Sir William Collins objects; probably the speaker had something of this sort in mind. In acknowledging the toast, the Master of the Rolls, Sir H. Cozens-Hardy, emphasised the point made by the proposer by saying that the long, unpronounceable, and unintelligible words used by some medical witnesses are the puzzle of juries and the distress of judges. Here the case for the use of simple and untechnical language is in our judgment much clearer. Scientifically speaking, great confusion would be caused by speaking of, say, "inflammation of the membrane between the skull and the brain" instead of the much more informative phrase "acute suppurative meningitis." The latter conveys to a medically trained hearer an exact picture of the condition and of the disease-process in action; the former conveys very little. Yet to a jury, or even to a judge, the simpler phrase conveys the truth and (as far as they can be expected to comprehend it) the whole truth, and nothing but the truth. Sir H. Cozens-Hardy added that lawyers have given up writing their opinions in Norman-French, but the parallel hardly holds to that extent. At any rate, the Medico-Legal Society has done well to ventilate the subject, even if only in the informality of after-dinner oratory, for it touches one aspect at least of a very important and interesting topic—the need and possibility of improvement in current medical standards of literature.

### The Problem of the Medical Treatment of School Children.

The London Education Committee on July 14 at its last meeting raised again that controversial problem which concerns the best means of securing the medical inspection on an organised wholesale plan, It is almost obvious that, granted the necessity for medical inspection on an organised wholesale plan, the removal or alleviation of the defects and ailments then discovered is equally necessary, and indeed is the only logical excuse for the preceding inspection. Merely to show up evils, and thereafter impotently flourish them, at great pains and much expense and without hope of achieving a remedy, needs considerable justification to enlist approval from plain common-sense ratepayers. But how to secure the necessary treatment of the defects discovered by the school medical officers at their routine inspection is a subject upon which all are not as yet agreed. The two main proposals which have so far divided the opinion of those interested each concern principles of great importance to education authorities, to hospital managers and subscribers, to medical practitioners and to the general ratepaying public. Moreover, they touch very vitally, as many think, the future character, almost as much as the future physique and health, of the lowest classes, in whose direct interest they are, of course, brought forward. We shall not for the moment do more than indicate the two methods at present under discussion; but we shall refer again in the near future to this problem. Firstly there are those who think that existing institutions, such as hospitals, are competent and appropriate to deal with the matter; and, secondly, there are those who would like to see the establishment of school clinics. So far, the Education Committee, by a majority, have adopted the "hospital" and rejected the "clinic" method. It is now reported that letters have been sent to 56 hospitals for eye, ear, and skin diseases; explaining the object which the Council has in view; asking how far their existing organisation would enable them to deal with increased numbers of child out-patients; and inquiring as to the conditions upon which the hospitals would be prepared to co-operate with the Council. Replies received from 39 hospitals seem to indicate that the great majority are in favour of this "hospital" scheme, and are moreover ready and willing to help in its furtherance, in return for a minimum of financial aid from the Council.

